# Identification of Prognostic Genes for Recurrent Risk Prediction in Triple Negative Breast Cancer Patients in Taiwan

**DOI:** 10.1371/journal.pone.0028222

**Published:** 2011-11-29

**Authors:** Lee H. Chen, Wen-Hung Kuo, Mong-Hsun Tsai, Pei-Chun Chen, Chuhsing K. Hsiao, Eric Y. Chuang, Li-Yun Chang, Fon-Jou Hsieh, Liang-Chuan Lai, King-Jen Chang

**Affiliations:** 1 Bioinformatics and Biostatistics Core, Center of Genomic Medicine, National Taiwan University, Taipei, Taiwan; 2 Department of Surgery, National Taiwan University Hospital, Taipei, Taiwan; 3 Institute of Biotechnology, National Taiwan University, Taipei, Taiwan; 4 Department of Statistics and Bioinformatics Science, Providence University, Taichung, Taiwan; 5 Department of Public Health, National Taiwan University, Taipei, Taiwan; 6 Graduate Institute of Biomedical Electronics and Bioinformatics, National Taiwan University, Taipei, Taiwan; 7 Department of Obstetrics and Gynecology, National Taiwan University Hospital, Taipei, Taiwan; 8 Graduate Institute of Physiology, National Taiwan University, Taipei, Taiwan; 9 Department of Surgery, Cheng Ching General Hospital, Taichung, Taiwan; Institute of Molecular and Cell Biology, Singapore

## Abstract

Discrepancies in the prognosis of triple negative breast cancer exist between Caucasian and Asian populations. Yet, the gene signature of triple negative breast cancer specifically for Asians has not become available. Therefore, the purpose of this study is to construct a prediction model for recurrence of triple negative breast cancer in Taiwanese patients. Whole genome expression profiling of breast cancers from 185 patients in Taiwan from 1995 to 2008 was performed, and the results were compared to the previously published literature to detect differences between Asian and Western patients. Pathway analysis and Cox proportional hazard models were applied to construct a prediction model for the recurrence of triple negative breast cancer. Hierarchical cluster analysis showed that triple negative breast cancers from different races were in separate sub-clusters but grouped in a bigger cluster. Two pathways, cAMP-mediated signaling and ephrin receptor signaling, were significantly associated with the recurrence of triple negative breast cancer. After using stepwise model selection from the combination of the initial filtered genes, we developed a prediction model based on the genes *SLC22A23*, *PRKAG3*, *DPEP3*, *MORC2*, *GRB7*, and *FAM43A*. The model had 91.7% accuracy, 81.8% sensitivity, and 94.6% specificity under leave-one-out support vector regression. In this study, we identified pathways related to triple negative breast cancer and developed a model to predict its recurrence. These results could be used for assisting with clinical prognosis and warrant further investigation into the possibility of targeted therapy of triple negative breast cancer in Taiwanese patients.

## Introduction

Breast cancer is the most common female solid tumor, and is among the top five leading causes of cancer-related death among Taiwanese women [1]. It is a heterogeneous disease that encompasses a spectrum of distinct phenotypes with disparate histopathological, clinical and molecular features. It can be classified into different subtypes on the basis of cellular morphology and the presence of several receptors, i.e., estrogen receptor (ER), progesterone receptor (PR), and human epidermal growth factor receptor 2 (ERBB2/HER2). Understanding its pathophysiology has led to the development of targeted therapies and improvement in clinical outcomes for patients. For example, for hormone receptor-positive breast cancer patients, hormone therapy has been approved as an adjuvant therapy, while for HER2-positive breast cancer, targeted therapy can be effective. However, one type of breast cancer, triple-negative breast cancer (TNBC), does not express ER, PR, and HER2. The signaling pathways and genes involved in TNBC have been studied for years, but there has been no significant progress toward adjuvant therapy. The prognosis of breast cancer is highly correlated with its subtype, and without adjuvant therapy, TNBC has the worst prognosis and of any breast cancer subtype [2]–[4]. The treatment of TNBC, therefore, remains a difficult challenge in clinical practice.

TNBC comprises approximately 10–16% of breast cancer cases [2], [5]. The main characteristic of TNBC is that it frequently affects younger patients, occurring predominantly in premenopausal women [6], [7]. The molecular mechanisms of TNBC still remain unclear, although their association with poor prognosis is thought to be due to aggressive biology and resistance to presently available endocrine therapies, agents targeting HER2 pathways, and standard cytotoxic chemotherapies.

Recent evidence supports the idea that the epidemiology and prognosis of breast cancer differs between races, most likely due to different genetic compositions [5], [8], [9]. Discrepancies in the prognosis of TNBC between Western and Asian populations in Taiwan were specifically noted [2]. However, few studies have investigated the genetic differences between breast cancers from Caucasian and Asian populations, let alone lower incidence of TNBC than other subtypes.

Since the advent of microarray chips, the mechanisms of breast cancer have been studied intensively, such that the subtype of breast cancer can be identified by its gene expression profile [10]. Breast cancer gene expression profiles have been identified across different microarray platforms by different research groups [11]. Several prediction models have been proposed, such as the 70-gene profile, two-gene ratio, or singular value decomposition, to predict lymph node metastasis [12]–[18]. The 70-gene profile for disease outcome prediction was a pioneering study, and has since been verified in several other studies [13]. It not only predicts outcomes effectively, but also outperforms other methods based on clinical parameters. Other studies using microarray gene expression profiles for clinical outcome prediction also provide satisfactory results [12], [14]–[17]. The use of microarray chips has proven to be a useful strategy to detect candidate genes and pathways involved in tumor progression.

The aforementioned success, however, has not been observed for TNBC patients, especially in the prediction of recurrence. Difficulties have arisen because of its relatively complex etiology, and because of the deficiency of TNBC samples. Yet, knowledge of the genes associated with recurrence of TNBC is desperately needed for designing prediction models and treatment strategies, possibly including targeted therapy.

This study used a Cox proportional hazards model to predict TNBC recurrence in a Taiwanese population. We compared the expression profiles of breast cancers from 185 Taiwanese patients to profiles from a Caucasian population [16]. The microarray results revealed differences in TNBC gene expression profiles in different ethnic groups. Pathway analysis showed that several canonical pathways, such as cAMP-mediated signaling and ephrin receptor signaling, are activated in association with recurrence in TNBC. Furthermore, six prognostic genes were identified for predicting the risk of recurrence of TNBC in Taiwanese patients.

## Materials and Methods

### Ethnic Statement

Written informed consent was acquired from all patients and/or guardians for the use of their tissue samples. This study was reviewed and approved by Research Ethics Committee of National Taiwan University Hospital.

### Sample collection

One hundred and eighty-five female breast cancer samples were collected at the National Taiwan University Hospital between 1995 and 2008. Breast tissue specimens were immediately snap-frozen in liquid N_2_ and stored at −80°C for RNA extraction. Similar numbers of samples in each subtype were included in this study; therefore the percentage of each subtype was not compatible with its prevalence.

### Isolation and amplification of total RNA for gene expression profiling

Total RNAs from the tissue specimens were extracted by TRIzol® reagent (Invitrogen, Carlsbad, CA) and subsequently purified with RNeasy® Mini Kits (Qiagen) according to the manufacturer's instructions. The integrity of RNA was determined using a 2100 BioAnalyzer (Agilent Technologies). RNA that had an RNA Integrity Number (RIN) >7.0 was used for microarray analysis. Total RNA (500 ng) was first reverse transcribed into cDNA by incorporating a T7 oligo-dT promoter primer prior to the generation of fluorescent Cy5-labeled cRNA using an Agilent Quick Amp Labeling Kit (Agilent Technologies). The labeled cRNA was purified using an RNeasy Mini Kit (Qiagen) and quantified using a NanoDrop ND-1000 instrument (Thermo Fisher Scientific). The common reference design was used where Universal Human Reference RNA (Stratagene, La Jolla, CA) was hybridized to every sample. Cy3- (reference) and Cy5-labeled (sample) cRNAs were co-hybridized to the Agilent Human 1Av2 oligo microarray using a Gene Expression Hybridization Kit (Agilent Technologies). All procedures were performed based on the manufacturer's protocols. The glass slides were scanned using an Agilent G2565BA microarray scanner. Raw data were collected using the Feature Extraction software (Agilent Technologies) and normalized by applying the rank consistency LOWESS normalization. Microarray data of this study are MIAME compliant, and have been submitted to the MIAME compliant GEO (Gene Expression Omnibus) database (accession number GSE33095).

### Data mining and statistical analysis

In order to investigate whether discrepancies exist in gene expression profiles of breast cancer between Caucasian and Asian populations, our data were combined with another microarray data with the same platform and analyzed together [16]. In order to make these two datasets comparable, the microarray data were processed as follows. First, due to the difference in versions of Agilent chips, only 8843 genes existing in both datasets were used for further analysis. Secondly, because the reference sample of van de Vijver's data was mixed samples of all breast cancer, not Universal Human Reference RNA, the expression value of each gene in our dataset was further normalized by its corresponding mean. Thirdly, differentially expressed genes were selected if they had a ≧4-fold change as compared to the median based on at least 15 samples. Fourthly, because the HER2 and PR staining status were not available, the expression levels of *ERBB2* and *PGR* were used for representing HER2 and PR status. After the HER2 and PR status was determined, the similarity of gene expression profiles between samples was assessed by average-linkage hierarchical clustering using Euclidean distance. The Database for Annotation, Visualization and Integrated Discovery (DAVID) was applied for significant genes differentiated by two-way ANOVA [19], [20]. Lastly, in order to examine their expression patterns in Asian populations, a list of basal-like characteristic genes in Caucasians was collected from the published literature [10], [11], [21], [22].

### Construction of the recurrence model for TNBC and pathway analysis

Among the 185 Taiwanese breast cancer patients, 51 were diagnosed with TNBC. After excluding those with distant metastasis upon diagnosis (n = 3), 48 patients with non-terminal stage TNBC were included for constructing the recurrence model. The time interval of recurrence was defined as the period between the surgery date and the date when recurrence was detected. In order to investigate the risk of recurrence among TNBC subjects, a Cox proportional hazards ratio model was applied to each gene. The coefficients in a Cox regression related to hazard. A positive coefficient in a Cox regression model indicated high risk of recurrence as expression level of gene increases; whereas a negative coefficient indicated low risk of recurrence as expression level of gene increases. Genes whose expression levels were associated with the time to recurrence of TNBC were selected at the significance level of 0.005 using Ingenuity Pathway Analysis (IPA; Ingenuity Systems Inc., Redwood City, USA). Furthermore, in order to select the most appropriate model for recurrence prediction, the Akaike information criterion (AIC) and Bayesian information criterion (BIC), measures of the goodness of fit of an estimated statistical model, were applied. Lastly, we chose the predictive model with the best score of model selection, and examined whether the model contained genes related to breast cancer.

### Cross-validation of the recurrence model by leave-one-out support vector regression

The support vector machine, a well-developed machine learning method, was used to train the recurrence model and cross-validate the results by leave-one-out. Briefly, support vector regression was done at two steps: first, training the recurrence model with current data; second, applying on a new dataset to predict the outcomes. The outcomes were cross-validated by using a single observation from the original sample as the validation data, and the remaining observations as the training data. This was repeated such that each observation in the sample was used once as the validation data.

## Results

### Clinical characteristics of samples

The clinical characteristics of samples used in this study are summarized in Table 1. Based on the result of immunohistochemistry (IHC) stains of ER, PR and HER2, there were 49 luminal A, 31 luminal B, 35 HER2, 51 triple negative, and 19 other types. All these patients received surgery, adequate chemotherapy and adjuvant therapy. Twenty-four of them were grade 1, 77 grade 2, and 71 grade 3. Among these patients, 33 were diagnosed as stage I, 84 as stage II, 58 as stage III, and 10 as stage IV. The median follow-up time was 4.15 years; 40 patients relapsed after their first surgery, and the rest (145 patients) did not experience recurrence by the end of this study.

**Table 1:** Summary statistics for patient profile (n = 185).

Clinical characteristics	Sample size (n)	Percentage	Sample size of TNBC (n)	Percentage of TNBC
Histological Types				
Luminal A	49	26%		
Luminal B	31	17%		
HER2	35	19%		
Triple negative	51	28%		
Others	19	10%		
Grade				
Grade 1	24	13%	0	0%
Grade 2	77	42%	15	29%
Grade 3	71	38%	32	63%
Missing data	13	7%	4	8%
Stage				
Stage I	33	18%	12	24%
Stage IIA	42	23%	18	35%
Stage IIB	42	23%	7	14%
Stage IIIA	29	16%	4	8%
Stage IIIB	3	2%	1	2%
Stage IIIC	26	14%	6	12%
Stage IV	10	5%	3	6%
Recurrence				
Yes	40	22%	14	27%
No	145	78%	37	73%

### Classification of tumor subtypes by gene expression profiling

**Figure 1:**
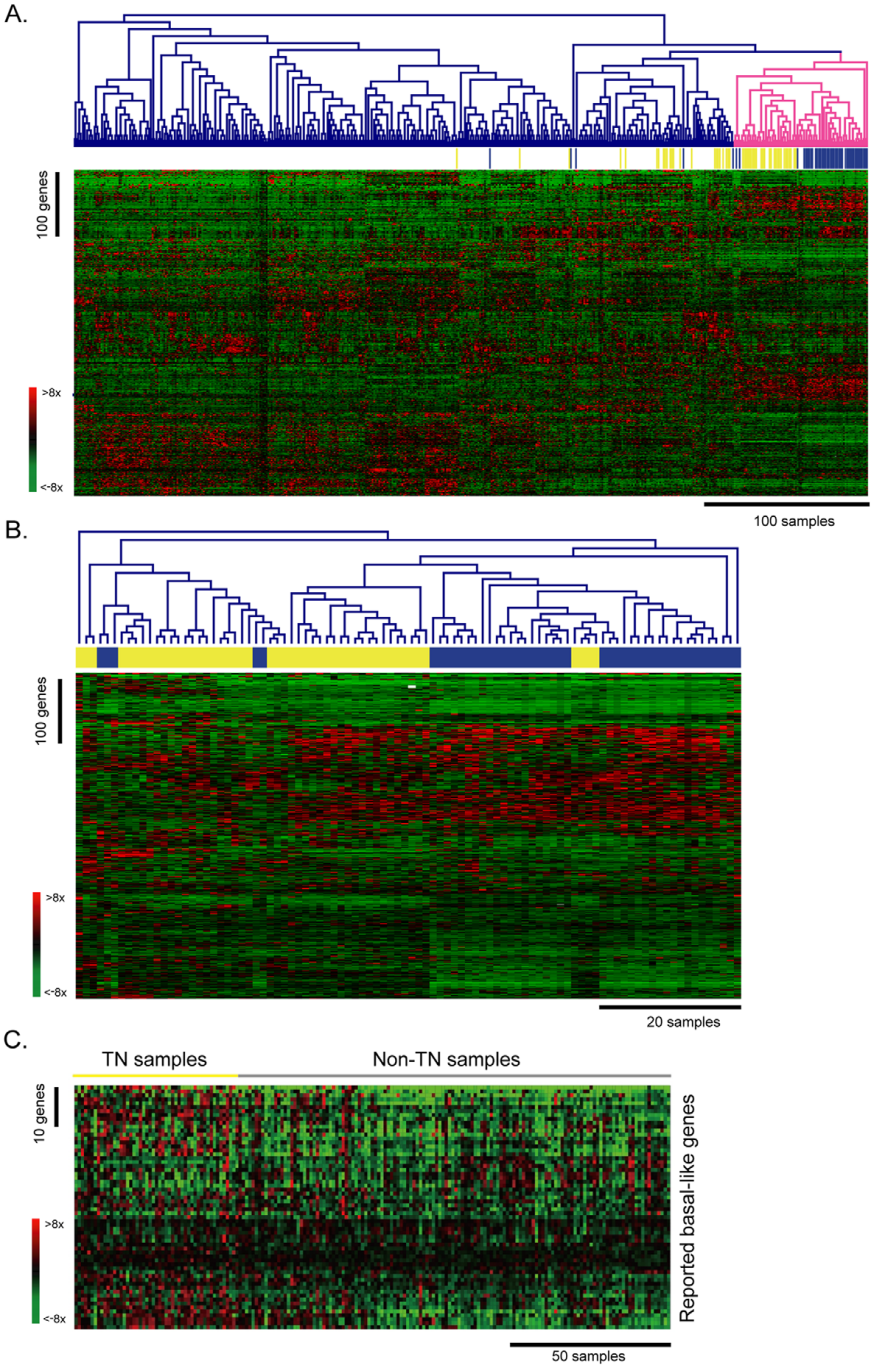
Gene expression profiles of Triple negative breast cancer differ between Caucasian and Asian populations in Taiwan. **A.** Hierarchical clustering of breast cancer samples from van de Vijver's study (n = 295) and this study (n = 185). Basal-like breast cancer samples in van de Vijver's study are marked with blue bars and those in this study are marked with yellow bars. The criterion of differentially expressed genes (n = 506) was that, among at least 15 samples, the gene had a ≧4-fold change as compared to the median. Expression values of genes were normalized by their respective means. Red indicates that the expression values were higher than average; green represents that the values were lower than average. **B.** Hierarchical clustering of basal-like breast cancer samples from van de Vijver's study (n = 49; blue) and this study (n = 64; yellow). **C.** The expression patterns of well-known basal-like genes in this study. The basal-like genes (n = 62) were obtained from previous studies [10], [11], [21], [22]. The triple negative (TN) samples are indicated with a yellow bar and the non-TN samples are indicated with a gray bar.

In order to investigate the differences in the expression profiles of breast cancers from Caucasian and Asian populations, we compared our microarray data with that of van de Vijver et al. [16]. To make these two datasets comparable, the data were processed as described in Materials and Methods. There were 506 differentially expressed genes, defined as a ≧4-fold change as compared to the median of at least 15 samples. Average-linkage hierarchical clustering using Euclidean distance was applied to the normalized expression values to evaluate the similarity of gene expression profiles between Caucasian and Asian populations. As shown in Figure 1A, TNBC samples in van de Vijver's study (blue bars) and those in this study (yellow bars) are in separate subclusters, but most of the TNBC samples were grouped in the bigger cluster (pink line). Although 29.8% of TNBC samples were scattered outside of the major group, these results still indicated that the characteristics of gene expression cannot only be used to distinguish TNBC from other cancer types, but also that differences in the expression profile of TNBC do exist between different ethnic groups. To emphasize the differences between the two ethnic groups, the dendrogram and heatmap were expanded by average-linkage clustering to show only the profiles of TNBC samples (Figure 1B). Similar procedures applied on another dataset GSE18229 (Figure S1), both results showed apparent differences between different races.

Next, statistical analysis using two-way ANOVA revealed that 66 genes had differential expression between the different races, but not across cancer subtypes. Pathway analysis using DAVID also revealed that these genes were involved in drug metabolism of fluorouracil and retinol metabolism, indicating that these pathways could account for the differences in ethnic groups but not in breast cancer subtypes (Table S1) [19], [20]. Furthermore, we collected 62 reported basal-like genes (Table S2) from Western populations, and examined their expression patterns in our samples [10], [11], [21], [22]. As shown in Figure 1C, no obvious up-regulation of these basal-like genes was observed in our TN samples, but they were all up-regulated in the samples from Western populations [10], [11], [21], [22]. These results showed that the expression profiles of TNBC are different in Caucasians and Asians.

### Pathway analysis and prediction model for recurrence

Since we knew that differences exist between Western and Eastern populations, we then focused on identifying genes associated with recurrence in our TNBC samples. There were 51 TNBC samples, and 48 of them were used for constructing a recurrence model because 3 TNBC patients had distant metastasis upon diagnosis. The time interval of recurrence was defined as the period between the surgery date and the date when recurrence was detected. In order to investigate the hazard of recurrence among TNBC subjects, a Cox proportional hazards model was constructed for each gene. There were 391 genes whose expression levels were significantly (*P<*0.005) associated with recurrence time in TNBC.

To further investigate which pathways were active in genes associated with recurrence of TNBC, Ingenuity Pathway Analysis was performed. Table 2 lists the canonical pathways in which genes associated with recurrence of TNBC were significantly enriched. The top two pathways were cAMP-mediated signaling and ephrin receptor signaling.

**Table 2:** Canonical pathways in which genes were associated with recurrence of TNBC.

Ingenuity Canonical Pathways	−log(*P*-value)	Gene No. (%*)	Genes#
cAMP-mediated Signaling	3.70	11 (6.83%)	*AKAP12, GNAI2, AKAP13, ADCY5, MAPK3, PDE1B, PRKAR1B, DUSP4, RAPGEF3, AKAP1, ATF2*
Ephrin Receptor Signaling	3.33	11 (5.61%)	*GNAI2, GRIN1, EPHA8, MAPK3, GRIN2D, ABL1, HRAS, ITGA3, PGF, EFNA1, ATF2*
Pancreatic Adenocarcinoma Signaling	3.09	8 (6.90%)	*TGFB1, MAPK3, ABL1, HBEGF, CDKN1B, E2F3, PGF, BCL2*
Amyotrophic Lateral Sclerosis Signaling	2.71	7 (6.25%)	*GRIN1, CACNA1E, GRIN2D, RAB5C, CAPN7, PGF, BCL2*
Cardiac β-adrenergic Signaling	2.66	8 (5.63%)	*AKAP12, AKAP13, CACNA1E, ADCY5, ADRBK2, PDE1B, PRKAR1B, AKAP1*
Chronic Myeloid Leukemia Signaling	2.66	7 (6.67%)	*TGFB1, MAPK3, HDAC7, ABL1, HRAS, CDKN1B, E2F3*
Circadian Rhythm Signaling	2.56	4 (1.14%)	*PER1, GRIN1, GRIN2D, ATF2*
Synaptic Long Term Potentiation	2.44	7 (6.19%)	*GRIN1, MAPK3, GRIN2D, PRKAR1B, HRAS, RAPGEF3, ATF2*
Cell Cycle: G1/S Checkpoint Regulation	2.37	5 (8.47%)	*TGFB1, HDAC7, ABL1, CDKN1B, E2F3*
Prostate Cancer Signaling	2.36	6 (6.25%)	*MAPK3, ABL1, HRAS, CDKN1B, BCL2, ATF2*

*Percentage of the number of differentially expressed genes in each canonical pathway.

#Differentially expressed genes in each canonical pathway.

Next, in order to establish an appropriate model to predict the probability of recurrence of TNBC, Cox proportional hazards models using different combinations of genes were applied. Considering that the total number of genes at *P*<0.005 level was enormous, genes at *P*<0.0005 level were used for establishing the prediction model. There were 35 genes with *P* values<0.0005 (Table 3).

**Table 3:** Genes whose expression levels were associated with the time to recurrence of TNBC using Cox proportional hazards regression.

Gene	Hazard*	*P*-value	Gene	Hazard	*P*-value
*FFAR2*	2.78	7.08×10^−6^	*LACRT*	2.00	3.20×10^−4^
*MKRN2*	0.17	1.97×10^−5^	*CCDC33*	3.23	3.36×10^−4^
*GPX3*	2.47	7.84×10^−5^	*SPRR1A*	2.49	3.48×10^−4^
*SCL22A23*	2.84	1.22×10^−4^	*MGC16385*	0.24	3.55×10^−4^
*INSM2*	2.60	1.31×10^−4^	*YPEL3*	3.43	3.59×10^−4^
*MRPS5*	0.38	1.33×10^−4^	*EPHA8*	2.08	3.75×10^−4^
*UFC1*	0.23	1.43×10^−4^	*HAS3*	2.89	3.76×10^−4^
*PRKAG3*	2.85	1.81×10^−4^	*HBEGF*	2.47	3.83×10^−4^
*PGF*	4.30	1.81×10^−4^	*CHST7*	3.34	3.89×10^−4^
*LPIN1*	0.19	1.83×10^−4^	*MRPS9*	0.27	3.99×10^−4^
*JUND*	3.40	1.92×10^−4^	*BCAM*	2.17	4.09×10^−4^
*DPEP3*	2.40	2.11×10^−4^	*GPR157*	2.65	4.19×10^−4^
*MORC2*	0.33	2.27×10^−4^	*ZNF285*	0.20	4.29×10^−4^
*SP2*	3.89	2.39×10^−4^	*MYO3A*	1.98	4.44×10^−4^
*GRB7*	2.12	2.41×10^−4^	*LCE1D*	2.30	4.53×10^−4^
*MAFK*	3.02	2.78×10^−4^	*TNFRSF4*	2.52	4.72×10^−4^
*FAM43A*	5.40	2.87×10^−4^	*C11orf56*	3.49	4.84×10^−4^
*EGFL7*	3.58	3.12×10^−4^			

*Hazard of each gene is the exponent of coefficient in a Cox regression.

The recurrence hazards of all possible combinations of 35 genes (34.3 billion combinations) were calculated using Cox proportional hazards models. The Akaike information criterion (AIC) was used to select the most appropriate model for recurrence prediction. The model with the smallest AIC was considered the best of 34.3 billion combinations. The top 10 best-fitted models are listed in Table 4. The first model, which contains *SLC22A23*, *PRKAG3*, *DPEP3*, *MORC2*, *GRB7*, and *FAM43A*, has not only the smallest AIC but also the smallest Bayesian information criterion (BIC). This model was further cross-validated by leave-one-out support vector regression, during which 91.7% accuracy with 81.8% sensitivity and 94.6% specificity was achieved. These results suggest that the six-gene model presented can be used to predict the recurrence of TNBC in Taiwanese patients.

**Table 4:** The top 10 prediction models for the recurrence of TNBC.

No.	Genes used in the Model	AIC*	BIC§
1	*SLC22A23*(−19.98)#,*PRKAG3*(51.51),*DPEP3*(−1.89), *MORC2*(−143.12),*GRB7*(141.32),*FAM43A*(167.24)	16.26	32.70
2	*SLC22A23*(−12.24),*PRKAG3*(33.01),*MORC2*(−88.76), *GRB7*(87.67),*FAM43A*(105.93),*BCAM*(0.52)	16.33	32.77
3	*PRKAG3*(33.07),*MORC2*(−66.15),*SP2*(−32.70), *GRB7*(48.66),*FAM43A*(57.79),*BCAM*(5.98)	17.09	33.54
4	*MKRN2*(−128.88),*GRB7*(47.29),*LACRT*(29.45), *SPRR1A*(62.98),*BCAM*(−110.68),*TNFRSF4*(41.51)	17.26	33.70
5	*FFAR2*(4.97),*PRKAG3*(10.68),*PGF*(−12.70), *MORC2*(−24.72),*GRB7*(18.91),*FAM43A*(26.48)	17.71	34.15
6	*PRKAG3*(24.00),*MORC2*(−52.48),*SP2*(−7.81), *GRB7*(47.24),*FAM43A*(49.48),*LCE1D*(−0.97)	17.74	34.18
7	*MKRN2*(−74.83),*PRKAG3*(52.61),*GRB7*(39.25), *MAFK*(−61.19),*SPRR1A*(8.38),*MRPS9*(−39.45)	17.77	34.21
8	*MKRN2*(−83.22),*MRPS5*(−35.24),*PRKAG3*(49.02), *DPEP3*(33.94),*CCDC33*(−53.64),*SPRR1A*(24.23)	17.89	34.33
9	*MKRN2*(−28.21),*MORC2*(−15.71),*GRB7*(20.86), *LACRT*(8.55),*SPRR1A*(20.30),*BCAM*(−25.95)	17.89	34.34
10	*MKRN2*(11.67),*PRKAG3*(46.49),*PGF*(−10.40), *MORC2*(−120.16),*GRB7*(114.51),*FAM43A*(125.69)	18.12	34.57

#The coefficient of each gene in Cox proportional hazard regression is shown in parentheses. A positive coefficient in a Cox regression model indicated high risk of recurrence as expression level of gene increases; whereas a negative coefficient indicated low risk of recurrence as expression level of gene increases.

*AIC: Akaike information criterion.

§BIC: Bayesian information criterion.

## Discussion

In this study we have shown that genetic profiling can be used not only to distinguish TNBC from other breast cancer types, but also to differentiate TNBCs derived from different ethnic groups. Using Cox proportional hazards models, genes and pathways that were associated with the recurrence of TNBC were identified. Moreover, we identified 6 genes (*SLC22A23*, *PRKAG3*, *DPEP3*, *MORC2*, *GRB7*, and *FAM43A*) which could be used to predict the recurrence of TNBC with 91.7% accuracy. These results could be used for assisting clinical prognosis and further investigation into targeted therapy of TNBC in Taiwanese.

The epidemiology and prognosis of breast cancer between different races were reported to be different [5], [8], [9]. Our results are the first to show the differences in genetic profiles of TNBC samples between Western and Asian populations in Taiwan. Furthermore, some of the most well-known genes for basal-like breast cancer, such as keratin 5 and keratin 17, were not even significantly different between our TN and non-TN samples [23]. These results indicate that biomarkers cannot be blindly used in different ethnic groups, and emphasize the importance of establishing biomarkers for TNBC in Asian populations.

Also, pathway analysis showed that 66 genes with differential expression patterns between races were involved in drug metabolism of fluorouracil and retinol metabolism. This finding may explain why some chemotherapy drugs have different effects on different ethnic groups. For example, several studies of Capecitabine, a prodrug of fluorouracil used for treating colorectal cancers, have shown different effects in different ethnic groups[24], [25]. One study demonstrated that thymidylate synthase, an important target for fluorouracil, may be expressed differently between Asian and Caucasian patients [24]. In addition, the hand-and-foot syndrome resulting from Capecitabine-associated toxicity may also display various patterns with different ethnic populations [25]. The other pathway that these genes were involved in was retinol metabolism. Since retinoic acid and retinol can enhance pigmentation of skin [26], it is not surprising that there is significantly different expression profiling between Asian and Caucasian populations, because the skin colors are different.

In our pilot study, we found that lymphovascular invasion, lymph node status, grade, nuclear pleomorphism, and tumor size were not associated with recurrence, but that age was mildly significantly associated (*P* = 0.0436) with recurrence. Therefore, we turned to gene expression to predict the recurrence of TNBC. Using Cox proportional hazards models, we identified 391 genes whose expression levels were significantly (*P<*0.005) associated with the recurrence time in TNBC. These genes were enriched in several pathways including cAMP-mediated signaling and ephrin receptor signaling. The ephrin receptor signaling pathway has been recognized in many studies of its tumor suppression in breast cancer[27], [28]. Our study confirmed the previous results and further indicated that ephrin receptor signaling is associated with the recurrence of TNBC. Other pathways could also be associated with the recurrence of TNBC, but further evaluation and studies are required. Previous studies have suggested the PARP1 inhibitor pathway for targeting TNBC, but it did not meet the significance cut-off in our pathway analysis [5]. This might be explained by the intrinsic differences between different races.

For developing the best model with the fewest genes, the Akaike information criterion (AIC) was used to evaluate the goodness of fit of the estimated combinations. After scrutinizing 34.3 billion of possibilities, the best Cox proportional hazards model for predicting the recurrence of TNBC contained the following 6 genes: *SLC22A23, PRKAG3, DPEP3, MORC2, GRB7*, and *FAM43A*. Among these 6 genes, the function of *SLC22A23 (*solute carrier family 22, member 23), *FAM43A* (family with sequence similarity 43, member A), and *DPEP3* (dipeptidase 3) remains unclear. *PRKAG3*, the protein kinase, AMP-activated, gamma 3 non-catalytic subunit, may play a role in regulating the energy metabolism of skeletal muscle [29], [30]. *MORC2* (MORC family CW-type zinc finger 2) was over-expressed in breast cancer tissue and in situ carcinoma as compared to adjacent normal breast tissue. However, its function in breast cancer remains unknown [31], [32]. *GRB7* (growth factor receptor-bound protein 7) could interact with some receptor tyrosine kinases and signaling molecules. Several studies have indicated that *GRB7* had an adverse prognostic effect on breast cancer outcomes and is associated with up-regulation of *GRB7* in breast cancer [32].

Because of the absence of sufficient clinical information from previously published studies, such as ER, PR, and HER2 status and the clinical outcomes of the patients, our data cannot be validated using an independent dataset. Furthermore, since the subjects in this study had different observation periods, the cross-validation was focused on predicting the possibility of recurrence. We cross-validated this model by using leave-one-out support vector regression. The accuracy of this model was 91.7%, the specificity for TNBC was 94.6%, and the sensitivity was 81.8% as compared to an average accuracy of 13.6% from one million permutations of any six-gene model. Yet, since the prediction model was established based on the TNBC patients in Taiwan, it is probably not applicable to Caucasian populations, since none of the genes found in our 6-gene prediction model were implicated by previous survival predictions determined in Caucasians using a 70-gene profile or a two-gene ratio [13], [14].

Because of the rarity of TNBC, the sample size of our study is small compared to other published studies. However, to our knowledge, this study has the largest sample size for TNBC in recent years. The presented 6-combination gene set, including *SLC22A23, PRKAG3, DPEP3, MORC2, GRB7*, and *FAM43A*, along with several significant pathways, might underlie the basic mechanism of the recurrence of TNBC, and points out a new avenue for further investigation.

## Supporting Information

Figure S1 Gene expression profiles of Triple negative breast cancer differ between Caucasian and Asian populations in Taiwan using dataset GSE18229. A. Triple negative breast cancer samples (marked by yellow: our samples and blue: western samples) were clustered regardless of different data sources. B. Only triple negative breast cancer samples were used in clustering (yellow: our samples, blue: western samples).
(TIF)

Table S1 Enriched pathways of differential expression genes between different races, but not in cancer subtypes, using DAVID.
(DOC)

Table S2 Gene list of basal-like associated genes from Western populations.
(DOC)

## References

[1] Bureau of Health Promotion DoH, ROC, Taiwan (2010). Cancer Registry Annual Report.

[2] Lin C, Chien SY, Chen LS, Kuo SJ, Chang TW (2009). Triple negative breast carcinoma is a prognostic factor in Taiwanese women.. BMC Cancer.

[3] Abd El-Rehim DM, Pinder SE, Paish CE, Bell J, Blamey RW (2004). Expression of luminal and basal cytokeratins in human breast carcinoma.. J Pathol.

[4] van de Rijn M, Perou CM, Tibshirani R, Haas P, Kallioniemi O (2002). Expression of cytokeratins 17 and 5 identifies a group of breast carcinomas with poor clinical outcome.. Am J Pathol.

[5] Cleator S, Heller W, Coombes RC (2007). Triple-negative breast cancer: therapeutic options.. The Lancet Oncology.

[6] Dent R, Trudeau M, Pritchard KI, Hanna WM, Kahn HK (2007). Triple-negative breast cancer: clinical features and patterns of recurrence.. Clin Cancer Res.

[7] Carey LA, Perou CM, Livasy CA, Dressler LG, Cowan D (2006). Race, Breast Cancer Subtypes, and Survival in the Carolina Breast Cancer Study.. JAMA.

[8] Kurebayashi J, Moriya T, Ishida T, Hirakawa H, Kurosumi M (2007). The prevalence of intrinsic subtypes and prognosis in breast cancer patients of different races.. Breast.

[9] Kim MJ, Ro JY, Ahn SH, Kim HH, Kim SB (2006). Clinicopathologic significance of the basal-like subtype of breast cancer: a comparison with hormone receptor and Her2/neu-overexpressing phenotypes.. Hum Pathol.

[10] Perou CM, Sorlie T, Eisen MB, van de Rijn M, Jeffrey SS (2000). Molecular portraits of human breast tumours.. Nature.

[11] Hu Z, Fan C, Oh D, Marron J, He X (2006). The molecular portraits of breast tumors are conserved across microarray platforms.. BMC Genomics.

[12] Huang E, Cheng SH, Dressman H, Pittman J, Tsou MH (2003). Gene expression predictors of breast cancer outcomes.. The Lancet.

[13] van't Veer L, Dai H, van de Vijver M, He Y, Hart A (2002). Gene expression profiling predicts clinical outcome of breast cancer.. Nature 415:.

[14] Ma X-J, Wang Z, Ryan PD, Isakoff SJ, Barmettler A (2004). A two-gene expression ratio predicts clinical outcome in breast cancer patients treated with tamoxifen.. Cancer Cell.

[15] Fan C, Oh DS, Wessels L, Weigelt B, Nuyten DSA (2006). Concordance among Gene-Expression-Based Predictors for Breast Cancer.. N Engl J Med.

[16] van de Vijver MJ, He YD, van't Veer LJ, Dai H, Hart AAM (2002). A Gene-Expression Signature as a Predictor of Survival in Breast Cancer.. N Engl J Med.

[17] Wang Y, Klijn JGM, Zhang Y, Sieuwerts AM, Look MP (2005). Gene-expression profiles to predict distant metastasis of lymph-node-negative primary breast cancer.. The Lancet.

[18] West M, Blanchette C, Dressman H, Huang E, Ishida S (2001). Predicting the clinical status of human breast cancer by using gene expression profiles.. Proceedings of the National Academy of Sciences of the United States of America.

[19] Dennis G, Sherman BT, Hosack DA, Yang J, Gao W (2003). DAVID: Database for Annotation, Visualization, and Integrated Discovery.. Genome Biol.

[20] Huang da W, Sherman BT, Lempicki RA (2009). Systematic and integrative analysis of large gene lists using DAVID bioinformatics resources.. Nat Protoc.

[21] Sorlie T, Perou CM, Tibshirani R, Aas T, Geisler S (2001). Gene expression patterns of breast carcinomas distinguish tumor subclasses with clinical implications.. Proceedings of the National Academy of Sciences of the United States of America.

[22] Sorlie T, Tibshirani R, Parker J, Hastie T, Marron JS (2003). Repeated observation of breast tumor subtypes in independent gene expression data sets.. Proceedings of the National Academy of Sciences of the United States of America.

[23] Peppercorn J, Perou CM, Carey LA (2008). Molecular Subtypes in Breast Cancer Evaluation and Management: Divide and Conquer.. Cancer Investigation.

[24] Marsh S, McLeod HL (2001). Thymidylate synthase pharmacogenetics in colorectal cancer.. Clin Colorectal Cancer.

[25] Saif MW, Sandoval A (2008). Atypical hand-and-foot syndrome in an African American patient treated with capecitabine with normal DPD activity: is there an ethnic disparity?. Cutan Ocul Toxicol.

[26] Sato K, Morita M, Ichikawa C, Takahashi H, Toriyama M (2008). Depigmenting mechanisms of all-trans retinoic acid and retinol on B16 melanoma cells.. Biosci Biotechnol Biochem.

[27] Zhang W, Zeng X, Briggs KJ, Beaty R, Simons B (2010). A potential tumor suppressor role for Hic1 in breast cancer through transcriptional repression of ephrin-A1.. Oncogene.

[28] Wykosky J, Gibo DM, Debinski W (2007). A novel, potent, and specific ephrinA1-based cytotoxin against EphA2 receptor, Äìexpressing tumor cells.. Molecular Cancer Therapeutics.

[29] Cheung PCF, Salt IP, Davies SP, Hardie DG, Carling D (2000). Characterization of AMP-activated protein kinase gamma-subunit isoforms and their role in AMP binding.. Biochemical Journal.

[30] Milan D, Jeon JT, Looft C, Amarger V, Robic A (2000). A mutation in PRKAG3 associated with excess glycogen content in pig skeletal muscle.. Science.

[31] Tripathi A, King C, de la Morenas A, Perry VK, Burke B (2008). Gene expression abnormalities in histologically normal breast epithelium of breast cancer patients.. Int J Cancer.

[32] Deblois G, Hall JA, Perry M-C, Laganiere J, Ghahremani M (2009). Genome-Wide Identification of Direct Target Genes Implicates Estrogen-Related Receptor {alpha} as a Determinant of Breast Cancer Heterogeneity.. Cancer Research.

