# Supplementary figures and images for: Identification of Prognostic Genes for Recurrent Risk Prediction in Triple Negative Breast Cancer Patients in Taiwan
Source: PLoS One. 2011 Nov 29;6(11):e28222. doi: 10.1371/journal.pone.0028222 (PMC3226667; doi:10.1371/journal.pone.0028222)

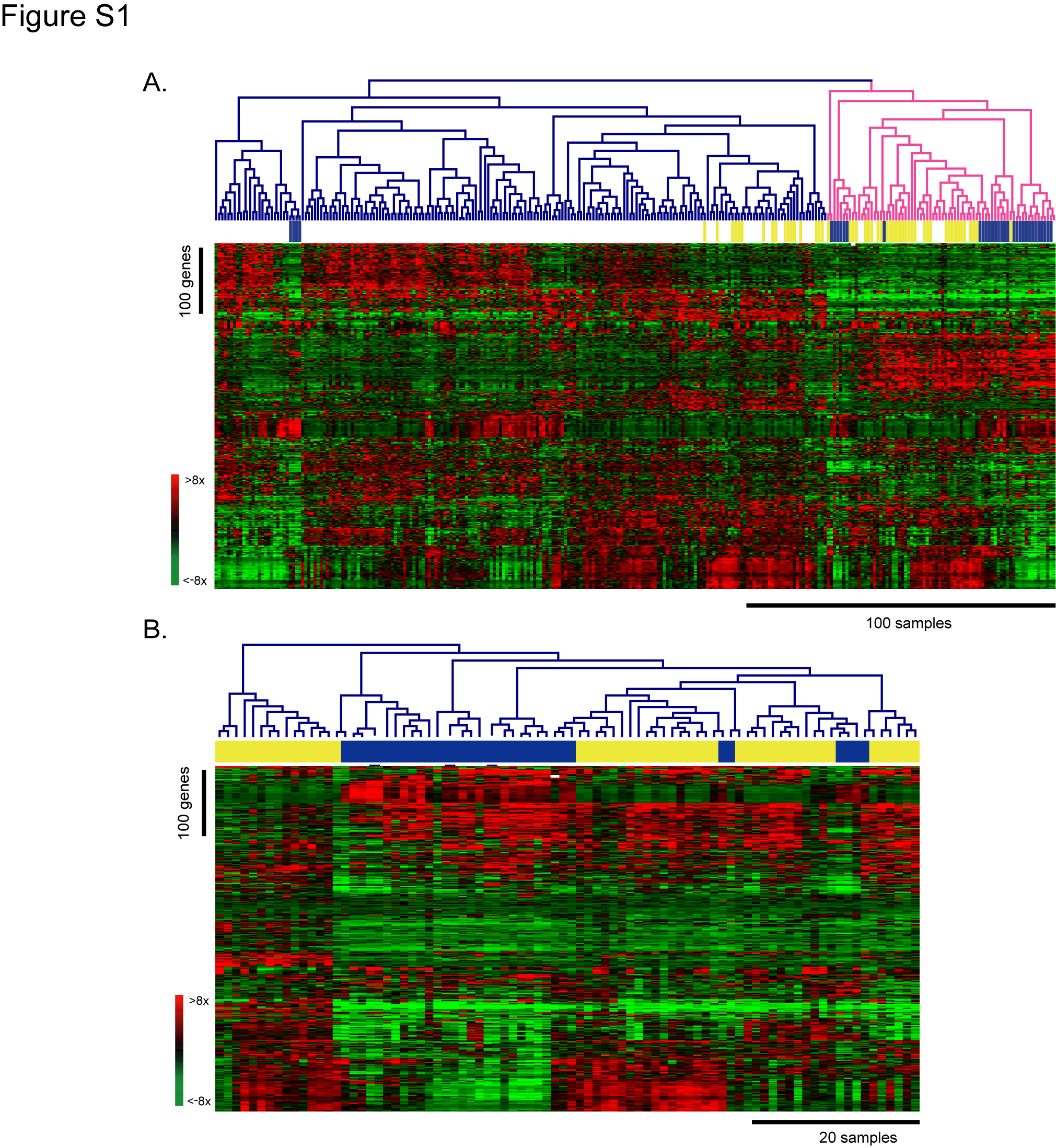

Supplement: Figure S1 — Gene expression profiles of Triple negative breast cancer differ between Caucasian and Asian populations in Taiwan using dataset GSE18229. A. Triple negative breast cancer samples (marked by yellow: our samples and blue: western samples) were clustered regardless of different data sources. B. Only triple negative breast cancer samples were used in clustering (yellow: our samples, blue: western samples). (TIF) [file pone.0028222.s001.tif]
