# Supplementary material for: Identification of Prognostic Genes for Recurrent Risk Prediction in Triple Negative Breast Cancer Patients in Taiwan
Source: PLoS One. 2011 Nov 29;6(11):e28222. doi: 10.1371/journal.pone.0028222 (PMC3226667; doi:10.1371/journal.pone.0028222)
Supplement: Table S1 — Enriched pathways of differential expression genes between different races, but not in cancer subtypes, using DAVID. (DOC) [file pone.0028222.s002.doc]

| Category | Term | Count | % | *P*-value | Genes |
| --- | --- | --- | --- | --- | --- |
| KEGG_PATHWAY | hsa00983:  Drug metabolism | 4 | 6.06 | 7.76×10^-4^ | *CYP2A13, UGT2B4, CYP2A7, UGT2B7* |
| KEGG_PATHWAY | hsa00830:  Retinol metabolism | 4 | 6.06 | 1.51×10^-3^ | *CYP2A13, UGT2B4, CYP2A7, UGT2B7* |

Table S1. Enriched pathways of differential expression genes between different races, but not in cancer subtypes, using DAVID.
