# Supplementary material for: Identification of Prognostic Genes for Recurrent Risk Prediction in Triple Negative Breast Cancer Patients in Taiwan
Source: PLoS One. 2011 Nov 29;6(11):e28222. doi: 10.1371/journal.pone.0028222 (PMC3226667; doi:10.1371/journal.pone.0028222)
Supplement: Table S2 — Gene list of basal-like associated genes from Western populations. (DOC) [file pone.0028222.s003.doc]

Table S2. Gene list of basal-like associated genes from Western populations.

| PROBE | GENE | PROBE | GENE |
| --- | --- | --- | --- |
| A_23_P24784 | *TNNI2* | A_23_P258456 | *FOXC1* |
| A_23_P16239 | *CNN1* | A_23_P11959 | *TGFB2* |
| A_23_P49928 | *KRT13* | A_23_P26843 | *SOX9* |
| A_23_P135748 | *TM4SF1* | A_23_P80598 | *CAPN7* |
| A_23_P22134 | *BNC1* | A_23_P153137 | *GALNT1* |
| A_23_P75589 | *CRYAB* | A_23_P12562 | *ANXA8* |
| A_23_P218040 | *KRT5* | A_23_P253038 | *DMD* |
| A_23_P203267 | *TRIM29* | A_23_P390504 | *FOXC1* |
| A_23_P215798 | *EGFR* | A_23_P48951 | *MFGE8* |
| A_23_P46090 | *LAD1* | A_23_P137672 | *CHI3L1* |
| A_23_P217570 | *CAPN6* | A_23_P216448 | *NFIB* |
| A_23_P49155 | *CDH3* | A_23_P123071 | *CAV2* |
| A_23_P134139 | *FABP7* | A_23_P52323 | *COL17A1* |
| A_23_P91230 | *SLPI* | A_23_P10121 | *SFRP1* |
| A_23_P59388 | *DST* | A_23_P7144 | *CXCL1* |
| A_23_P96149 | *KRT17* | A_23_P12082 | *CHI3L2* |
| A_23_P381378 | *CAPN7* | A_23_P415510 | *LAD1* |
| A_23_P110253 | *KIT* | A_23_P125233 | *CNN1* |
| A_23_P212905 | *CXCL1* | A_23_P94501 | *ANXA1* |
| A_23_P414793 | *CP* | A_23_P306105 | *GALNT1* |
| A_23_P209449 | *FZD7* | A_23_P255884 | *GSN* |
| A_23_P37727 | *CX3CL1* | A_23_P337070 | *S100A2* |
| A_23_P254242 | *PTPRK* | A_23_P201636 | *LAMC2* |
| A_23_P139704 | *DUSP6* | A_23_P20336 | *DPYSL2* |
| A_23_P144976 | *PIK3R1* | A_23_P52761 | *MMP7* |
| A_23_P44724 | *CSRP2* | A_23_P313591 | *KRT7* |
| A_23_P205509 | *MMP14* | A_23_P381945 | *KRT7* |
| A_23_P303085 | *PTN* | A_23_P59375 | *ID4* |
| A_23_P18372 | *B3GNT5* | A_23_P117882 | *CRABP1* |
| A_23_P254924 | *PTPRK* | A_23_P89780 | *LAMA3* |
| A_23_P71170 | *TRPV6* | A_23_P134213 | *PTN* |
